# Do risk factors at the time of hospital admission differ by sex for in-hospital mortality from coronavirus disease 2019 (COVID-19)?

**DOI:** 10.1017/ash.2021.223

**Published:** 2021-11-25

**Authors:** Mamta Sharma, Ashish Bhargava, Susanna M. Szpunar, Leonard B. Johnson, Louis D. Saravolatz

**Affiliations:** Ascension St John Hospital, Detroit, Michigan

## Abstract

**Background:**

Sex-disaggregated data for coronavirus disease 2019 (COVID-19) reported higher hospitalized fatality rates among men than women.

**Objective:**

To determine whether the risk factors for in-hospital mortality from COVID-19, present at the time of hospital admission, differed by patient sex.

**Design and setting:**

Single-center, retrospective cohort study at a tertiary-care urban academic center.

**Methods:**

We reviewed the electronic medical records of patients positive for COVID-19 via qualitative polymerase chain reaction (PCR) assay, admitted between March 8 and June 14, 2020. Patients were stratified by sex to assess the association of variables present on admission with in-hospital mortality.

**Results:**

The overall inpatient case fatality rate (CFR) was 30.4% (172 of 565). The CFR among male patients was higher than among female patients: 99 (33.7%) versus 73 (26.9%), respectively (*P* = .08). Among males, comorbid conditions associated with in-hospital mortality were chronic pulmonary disease (*P* = .02) and connective tissue disease (*P* = .03). Among females, these comorbid conditions were congestive heart failure (*P* = .03), diabetes with complication (*P* = .05), and hemiplegia (*P* = .02). Variables that remained independently associated with death in males included age >70 years, public insurance, incremental increase in quick sepsis-related organ failure assessment (qSOFA) and C-reactive protein (CRP), lymphocytopenia, and thrombocytopenia. Among females, variables that remained independently associated with mortality included public insurance, incremental increase in Charlson weighted index of comorbidity (CWIC) score, qSOFA, and CRP.

**Conclusions:**

Risk factors for in-hospital mortality by sex included public insurance type, incremental increase in qSOFA and CRP in both sexes. For male patients, older age, lymphocytopenia and thrombocytopenia were also associated with mortality, whereas a higher Charlson score was associated with in-hospital mortality in female patients.

Coronavirus disease 2019 (COVID-19) is a global pandemic that has resulted in >200 million infections and >4 million deaths worldwide.^1^ Early studies from China suggested sex differences, with higher hospitalization and mortality among men than females.^2,3^ These findings were confirmed with sex-disaggregated data for COVID-19 from 140 countries, which accounted for similar numbers of cases in women compared to men but higher hospitalized case-fatality rates among men than among women.^4^

Men and women differ in both innate and adaptive immune responses. Severe acute respiratory syndrome coronavirus 2 (SARS-CoV-2) uses the SARS-CoV receptor angiotensin-converting enzyme 2 (ACE-2) as an entry receptor, with virus entry enhanced by cellular transmembrane serine protease 2 (TMPRSS-2). ACE-2 is an X chromosome–encoded gene that is downregulated by estrogens, whereas TMPRSS-2 is regulated by androgen receptor signaling.^5–8^ This factor might lead to increased ACE-2 expression in men and decreased expression in women.

The high burden of illness and high case-fatality rate in men with COVID-19 may be driven in part by the strong affinity of the virus for ACE-2, leading to virus entry and multisystem illness in pulmonary, gut, renal, cardiac, and central nervous systems. These effects may be modified by differential engagement in daily activities and social behaviors. Men are more likely to engage in poor health behaviors such as smoking.^9^ Women are more likely to follow isolation and mask-wearing recommendations,^10^ and more women than men get tested for SARS-CoV-2.

Understanding the clinical risk factors and laboratory biomarkers associated with severe^11^ and fatal COVID-19 by sex in a community setting may allow early interventions to help mitigate adverse outcomes. Among hospitalized patients with COVID-19, we assessed whether risk factors for in-hospital mortality that were present at the time of admission differed by sex.

## Methods

### Study setting and design

We conducted a single-center, historical cohort study at a 776-bed tertiary-care urban academic medical center. The study was approved by the Ascension St John Hospital Institutional Review Board. Hospitalized adult patients with confirmed COVID-19 (positive real-time reverse-transcriptase-polymerase-chain-reaction (RT-PCR) assay of a nasopharyngeal swab) from March 8 to June 14, 2020, were included.

### Data collection

Data were collected from the electronic medical record for all the patients meeting inclusion criteria. Collected clinical information included demographics, presence of comorbid conditions, Charlson weighted index of comorbidity (CWIC),^12^ presenting symptoms, initial vital signs, admission laboratory and radiological findings, and outcome variables including need for mechanical ventilation, intensive care unit admission, and discharge disposition.

Patients were stratified by sex to determine whether the factors associated with in-hospital mortality differed by the sex of the patient.

### Statistical analysis

Statistical analysis was performed using SPSS version 28.0 software (IBM, Armonk, NY). Descriptive statistics were generated to characterize the study group. Continuous variables were described as the mean with standard deviation or median with interquartile range, and categorical variables were described as frequency distributions. Univariable analyses were conducted using the Student *t* test, the Mann-Whitney U test, and χ^2^ analysis. Variables that were significant or nearly significant (*P* < .09) predictors of mortality were then entered into a multivariable logistic regression model using a forward likelihood ratio algorithm. For comorbidities, the CWIC score was used instead of individual comorbid conditions. When 2 variables were measuring the same underlying factor, the variable with the highest univariable measure of association was used in the model. Results from the regression are reported as odds ratios with 95% confidence intervals. All *P* values are 2-sided.

## Results

In total, 565 adult hospitalized patients with confirmed SARS-CoV-2 infections were included.

Risk factors for overall mortality have been previously described in detail.^13^ The overall inpatient case fatality rate (CFR) was 30.4% (172 of 565). There were no differences in mean age of the cohort by sex, 64.3 years (SD, 17.8) years versus 64.5 years (SD, 14.6 years for females and males, respectively; *P* = .89. Women had a significantly higher mean BMI than men: 33.7 kg/m^2^ (SD, 9.5) versus 30.4 kg/m^2^ (SD, 8.3) (*P* < .0001). The CFR among male patients was higher than among female patients: 99 (33.7%) versus 73 (26.9%), respectively (*P* = .08). However, the difference only approached statistical significance.

### Univariable predictors of in-hospital mortality among both males   and females

Age >70 years, higher CWIC scores, public insurance, and admission from nursing facilities were associated with higher inpatient mortality (Tables 1 and 2). Patients who died also had significantly higher mean quick sepsis-related organ failure assessment (qSOFA) scores at the time of hospital admission than those who survived.

**Table 1.** Sex-Stratified Univariable for Categorical Variables and χ^2^ Test Analysis of Predictors of In-hospital Mortality From COVID-19 Variable

	Males				Females			
	Discharged (N = 195), No. (%)	Died (N = 99), No. (%)	OR (95% CI)	*P* Value	Discharged (N = 198), No. (%)	Died (N = 73), No. (%)	OR (95% CI)	*P* Value
**Age**								
<70 y	136 (69.7)	44 (44.4)			124 (62.6)	34 (46.6)		
>70 y	59 (30.3)	55 (55.6)	2.9 (1.7–4.8)	<.0001	74 (37.4)	39 (53.4)	1.9 (1.1–3.3)	.02
**Race**								
White	35 (18.7)	30 (30.6)			38 (19.4)	12 (16.4)	1.2 (0.59–2.49)	
Black	152 (81.3)	68 (69.4)	0.52 (0.30–0.92)	.02	156 (79.6)	60 (82.2)	1.2 (0.59–2.49)	.59
**BMI (kg/m** ^2^ **)**								
<25	30 (15.9)	34 (34.7)			28 (14.2)	16 (21.9)		
>25	159 (84.1)	64 (65.3)	0.4 (0.2–0.6)	<.0001	169 (85.8)	57 (78.1)	0.6 (0.3–1.2)	.13
**Insurance type**								
Commercial	63 (32.1)	8 (8.2)			52 (26.3)	8 (11.0)		
Public	133 (67.9)	90 (91.8)	5.4 (2.5–11.8)	<.0001	146 (73.7)	65 (89.0)	2.9 (1.3–6.4)	.007
**Admission source**								
Home	148 (75.9)	54 (54.5)			149 (75.3)	45 (61.6)		
Nursing home	47 (24.1)	45 (45.5)	2.6 (1.6–4.4)	<.0001	49 (24.7)	28 (38.4)	1.9 (1.1–3.4)	.03
**Comorbidities, no. (%)**								
Congestive heart failure	28 (14.4)	21 (21.2)	1.6 (0.9–3.0)	.14	19 (9.6)	14 (19.2)	2.2 (1.1–4.7)	.03
Dementia	20 (10.3)	28 (28.3)	3.5 (1.8–6.5)	<.0001	28 (14.1)	19 (26.0)	2.1 (1.1–4.1)	.02
Chronic pulmonary disease	31 (15.9)	27 (27.3)	2.0 (1.1–3.6)	.02	55 (27.8)	16 (21.9)	0.7 (0.4–1.4)	.33
Connective tissue disease	1 (0.5)	4 (4.0)	8.1 (0.9–73.7)	.03	5 (2.5)	1 (1.4)	0.5 (0.1–4.7)	.57
Diabetes without complication	54 (27.7)	32 (32.3)	1.2 (0.7–2.1)	.41	52 (26.3)	16 (21.9)	0.8 (0.4–1.5)	.46
Diabetes with complication	24 (12.3)	10 (10.1)	0.8 (0.4–1.7)	.58	18 (9.1)	13 (17.8)	2.2 (1.0–4.7)	.05
Hemiplegia	10 (5.1)	9 (9.1)	1.9 (0.7–4.7)	.19	9 (4.5)	9 (12.3)	3.0 (1.1–7.8)	.02
Renal disease	34 (17.5)	22 (22.2)	1.3 (0.7–2.5)	.33	30 (15.2)	14 (19.2)	1.3 (0.7–2.7)	.43
Malignancy	8 (4.1)	9 (9.1)	2.3 (0.9,6.3)	.08	10 (5.1)	7 (9.6)	2.0 (0.7–5.5)	.17
Hypertension	140 (71.8)	79 (79.8)	1.6 (0.9–2.8)	.14	142 (71.7)	57 (78.1)	1.4 (0.7–2.7)	.29
Obesity	91 (48.1)	36 (36.7)	0.63 (0.4–1.03)	.07	125 (63.5)	48 (65.8)	1.1 (0.6–1.9)	.72
**Vitals, no. (%)**								
**Systolic BP on admission**								
>100 mm Hg	182 (93.3)	85 (85.9)			183 (92.4)	62 (84.9)		
<100 mm Hg	13 (6.7)	14 (14.1)	2.3 (1.04–5.1)	.04	15 (7.6)	11 (15.1)	2.2 (0.9–5.0)	.06
**Respiratory rate**								
<22 breaths/minute	111 (56.9)	35 (35.4)			106 (53.5)	24 (32.9)		
≥22 breaths/minute	84 (43.1)	64 (64.6)	2.4 (1.5–4.0)	<.0001	92 (46.5	49 (67.1)	2.4 (1.3–4.1)	.003
**Symptoms, no. (%)**								
Fever	106 (54.9)	50 (51.5)	0.9 (0.5–1.4)	.59	121 (61.7)	37 (50.7)	0.6 (0.4–1.1)	.10
Altered mental status	41 (21.0)	45 (45.5)	3.1 (1.9–5.3)	<.0001	42 (21.2)	31 (42.5)	2.7 (1.5–4.9)	<.0001
**Laboratory findings on admission, no. (%)**								
Leukopenia	21 (10.8)	10 (10.1)	0.9 (0.4–2.1)	.86	23 (11.6)	6 (8.2)	0.7 (0.3–1.7)	.42
Lymphocytopenia	99 (50.8)	66 (68.0)	2.1 (1.2–3.4)	.005	79 (40.3)	38 (52.1)	1.6 (0.9–2.8)	.08
Thrombocytopenia	37 (19.2)	31 (31.3)	1.9 (1.1–3.4)	.02	30 (15.2)	19 (26.4)	2.0 (1.04–3.8)	.04
Elevated AST (>40 U/L)	101 (55.5)	65 (68.4)	1.7 (1.03–2.9)	.04	90 (48.1)	43 (66.2)	2.1 (1.2–3.8)	.01
Low serum proteins (<6.2 gm/dl)	12 (6.4)	12 (12.6)	2.1 (0.9–4.9)	.07	14 (7.3)	11 (15.5)	2.3 (1.0–5.4)	.04
Low serum albumin (<3.5 gm/dL)	78 (41.5)	65 (68.4)	3.1 (1.8–5.1)	<.0001	70 (36.3)	45 (63.4)	3.0 (1.7–5.4)	<.0001
Elevated LDH (>500 U/L)	28 (24.1)	24 (42.1)	2.3 (1.2–4.5)	.02	18 (15.1)	14 (34.1)	2.9 (1.3–6.6)	.009

Note. OR, odds ratio; CI, confidence interval; BMI, body mass index; AST, aspartate aminotransferase; LDH, lactate dehydrogenase.

Pre-existing renal disease was defined as chronic dialysis, history of renal transplant, uremic syndrome, or a creatinine >3 mg/dL on prior admissions. Malignancy was included if active or treated in the last five years. Leukopenia and lymphocytopenia

were defined as white blood cell counts of <4.0 × 10^9^/L and an absolute lymphocyte count <1.0 × 10^9^/L. Thrombocytopenia was defined as a platelet count <150 × 10^9^/L.

**Table 2.** Sex-Stratified Univariable Analysis of Predictors of In-Hospital Mortality From COVID-19 for Continuous Variables, Student *t* Test and Mann-Whitney *U* Test

Variable	Males			Females		
	Discharged (N = 195)	Died (N = 99)	*P* Value	Discharged (N = 198)	Died (N = 73)	*P* Value
Age at admission, mean y ± SD	61.3 ± 14.6	70.9 ± 12.4	<.0001	62.3 ± 18.1	69.7 ± 16.0	.003
BMI, mean kg/m^2^± SD	30.9 ± 7.5	29.4 ± 9.5	.15	33.8 ± 9.1	33.3 ± 10.5	.70
Median CWIC score (IQR)	1 (0, 2)	2 (1, 3)	<.0001	1 (0, 2)	2 (1, 3)	<.0001
No. of comorbidities, mean ± SD	2.6 ± 1.7	3.2 ± 1.6	.004	2.8 ± 1.8	3.3 ± 1.6	.04
Symptom onset to admission days, mean ± SD	5.6 ± 4.5	4.4 ± 3.9	.03	5.4 ± 3.5	5.2 ± 4.4	.71
qSOFA score, Mean ± SD	0.7 ± 0.7	1.2 ±0.8	<.0001	0.8 ±0.7	1.3 ± 0.8	<.0001
WBC (10^9^/L) on admission, Mean ± SD	7.6 ± 3.8	8.7 ± 3.8	.02	7.5 ± 3.7	8.6 ± 4.9	.07
Absolute lymphocytes (10^9^/L) on admission, mean ± SD	1.1 ± 0.7	0.9 ± 0.5	.001	1.2 ± 0.6	1.1 ± 0.9	.46
CRP (mg/L), mean ± SD	98.7 ± 79.8	139.9 ± 94.4	<.0001	77.6 ± 64.8	115.6 ± 88.3	.002
Creatinine, median mg/dL (IQR)	1.3 (1.0)	1.6 (1.3)	.05	1.0 (0.7)	1.3 (1.1)	<.0001

Note. SD, standard deviation; BMI, body mass index; CWIC, Charlson weighted index of comorbidity; qSOFA, quick sepsis-related organ failure assessment; WBC, white blood count; CRP, C-reactive protein; IQR, interquartile range.

Irrespective of sex, patients who died during the hospitalization versus those who survived were more likely to have thrombocytopenia, hypoalbuminemia, elevated aspartate aminotransferase (AST) level, elevated C-reactive protein (CRP) level, and elevated creatinine level on admission (Table 1 and 2).

### Univariable predictors of in-hospital mortality in males

Among men, black race and body mass index (BMI) >25 (kg/m^2^) were associated with in-hospital mortality. Comorbid conditions that were associated with death were chronic pulmonary disease and connective tissue disease.

In terms of vital signs, systolic blood pressure <100 mmHg and fever within 24 hours of admission were more prevalent among men who died than those who survived, respectively (Table 1). The mean duration of symptoms prior to hospitalization was significantly shorter in men who died than in those who survived. The prevalence of lymphocytopenia was higher among men with in-hospital mortality than those who were discharged (Table 2).

### Multivariable analysis of in-hospital mortality in males

For multivariable logistic regression, variables initially entered included age >70 years, race, BMI, CWIC, insurance type, hospital admission source, lymphocytopenia on admission, thrombocytopenia on admission, total protein on admission, albumin on admission, CRP, serum creatinine and qSOFA score on admission. Variables that remained independently associated with death in men included age >70 years, public insurance, incremental increase in qSOFA, and CRP, lymphocytopenia, and thrombocytopenia (Table 3).

**Table 3.** Sex-Stratified Multivariable Logistic Regression of Predictors of In-Hospital Mortality From COVID-19

Variable	Odds Ratio	95% CI	*P* Value
**Analysis for Males**			
Age>70	2.04	1.1–3.9	.03
Insurance Type	2.9	1.2–7.1	.02
qSOFA	1.8	1.2–2.7	.005
Lymphocytopenia	2.003	1.1–3.6	.022
Thrombocytopenia	2.2	1.1–4.5	.02
CRP on admission	1.004	1.001–1.008	.02
**Analysis for females**			
Insurance Type	3.1	0.99–9.8	.05
Charlson score	1.2	1.1–1.4	.005
qSOFA	1.9	1.2–3.0	.009
CRP on admission	1.007	1.002–1.011	.005

Note. CI, confidence interval; qSOFA, quick sepsis-related organ failure assessment; CRP, C-reactive protein.

### Univariable predictors of in-hospital mortality in females

Among women, comorbid conditions associated with in-hospital mortality included congestive heart failure, diabetes with complications and hemiplegia (Table 1). There was a higher prevalence of low serum protein (<6.2 gm/dL) in females with in-hospital mortality compared to those who were discharged (Table 1).

### Multivariable analysis of in-hospital mortality in females

For multivariable logistic regression, variables initially entered included age >70 years, race, BMI, CWIC, insurance type, hospital admission source, lymphocytopenia on admission, thrombocytopenia on admission, total protein on admission, albumin on admission, AST > 40U/L, CRP, serum creatinine on admission, and qSOFA score on admission. Variables that remained independently associated with in-hospital mortality included public insurance, incremental increase in CWIC score, qSOFA, and CRP (Table 3).

## Discussion

Although numerous studies have reported differences in case-fatality rates by sex, our objective was to determine whether the risk factors for death from COVID-19 differed by the sex of the patient.

In our study, independent predictors for in-hospital mortality common to both sexes were public insurance, higher qSOFA score, and elevated CRP at the time of admission. However, older age, presence of lymphocytopenia, and presence of thrombocytopenia at the time of hospital admission were significant predictors for in-hospital mortality among men only whereas higher CWIC score remained the only independent predictor specific to women.

Public insurance type was associated with in-hospital mortality. For most working-aged adults, eligibility for public insurance is primarily dependent on age. In a national survey data study, Sorlie et al^14^ reported increased mortality in individuals with public insurance as compared to those with employer-provided insurance. After adjustment for age and income, persons with Medicare and Medicaid had the highest mortality compared with those who had employer-provided insurance, with relative risks generally >2.^14^

The qSOFA score was introduced by Sepsis-3 task force as a tool to assist in the early identification of patients at risk of sepsis. Patients with a qSOFA score of ≥2 had a mortality rate of 24% compared to 3% among patients with a qSOFA score of <2.^15^ We previously showed that bedside qSOFA score at the time of hospitalization can predict in-hospital mortality among adults aged ≤65 years with COVID-19, for patients with qSOFA scores of 0, 1, 2, and 3 at the time of hospital admission, the mortality rates were 8.7%, 19.7%, 41.7%, and 60%, respectively.^16^ In our present study, men and women both demonstrated a linear trend of increase in mortality with increasing qSOFA score (*P* < .001). From multivariable analysis, every 1-unit increase in qSOFA score increased the risk of death by 80% in men and 90% in women.

CRP is an acute-phase protein that is stimulated by the release of cytokines. CRP level upon admission has been associated with severe infection. In a previous study, we demonstrated that every 1-unit increase in CRP increased the risk of severe disease by 0.06%.^17^ We have previously reported that after controlling for other variables, every unit increase in CRP increased the risk of death by 0.4% among admitted black patients.^18^ A meta-analysis also revealed elevated CRP leading to poor outcomes in COVID-19 patients (OR, 11.97; 95% CI 4.97–28.8; *P* < .001). Both male and female patients who died of COVID-19 displayed significantly higher CRP concentrations compared to survivors.^19^ There seems to be a vigorous proinflammatory cytokine and chemokine response to the virus leading to apoptosis of endothelial cells, and epithelial cells which damages the pulmonary microvascular barriers and causes vascular leakage and alveolar edema, leading to ARDS. A positive association between CRP concentrations and lung lesions was reported in COVID-19 patients. From multivariable analysis, every unit increase in CRP increased the risk of death by 0.4% in men compared to 0.7% for women.

Early US epidemiologic data suggest that the case fatality is highest in persons aged ≥85 years (range, 10%–27%), followed by 3%–11% for those aged 65–84 years.^20^ Hazard ratios increase with age, increasing from 2.40 in those aged >60 years to 20.61 in individuals aged >80 years in the Open SAFELY study.^21^ Poorer outcomes in the elderly may result from immune dysregulation leading to insufficient immunologic or excess inflammatory responses. Age ≥70 years was associated with mortality in men only in our present study.

Thrombocytopenia in critically ill patients usually suggests serious organ malfunction or physiologic decompensation, often evolving toward disseminated intravascular coagulation. Thrombocytopenia might be a risk factor for COVID-19 progressing into a more severe state.^22^ In a meta-analysis by Lippi et al,^23^ a more substantial drop in platelets was observed in nonsurvivors (*P* < .001). In an earlier study among adults aged ≤65 years with COVID-19, 37.5% of nonsurvivors had thrombocytopenia on presentation, compared to 14% of survivors.^16^ In our study, thrombocytopenia was associated with mortality in men only.

Tan et al^24^ reported lymphocytopenia to be an effective indicator for severe COVID-19. They postulated that the virus might directly destroy lymphocytes and lymphatic organs. Injured alveolar epithelial cells could induce the infiltration of lymphocytes, leading to persistent lymphopenia and worsening ARDS. Another study reported decreased absolute T-cell counts, especially CD8^+^ T cells, instead of B cell or natural killer cells to be associated with severe infection.^25^ A systematic review and meta-analysis showed that patients who died had lower lymphocyte counts (mean difference, −395.35 μL; 95% CI, −165.64 to −625.07; *P* < .001).^26^ We previously showed that lymphocytopenia at the time of hospital admission was an independent predictor for in-hospital mortality. In our cohort, lymphocytopenia was more frequent in patients who died versus those who survived (62.5% vs 46.9%).^13^ In our present cohort, lymphocytopenia was associated with mortality in men only.

Similar to the findings of Eboni et al,^27^ a higher CWIC score was an independent predictor of mortality in our study in women only. Congestive heart failure, hemiplegia, and diabetes with complications were independent risk factors for death among women in our study cohort. We also previously found that a higher CWIC score was an independent predictor of mortality. Congestive heart failure, dementia, hemiplegia, malignancy, and severe liver disease were associated with mortality. In Open SAFELY, study comorbidities associated with a higher risk of COVID-19–related death, included diabetes, severe asthma, respiratory disease, chronic heart disease, liver disease, stroke, dementia, other neurological diseases, reduced kidney function, autoimmune diseases, and other immunosuppressive conditions.

This study had several limitations. It was limited by the sample size. This study was from a single institution caring predominantly for black patients who required hospitalization, which makes generalization difficult. Because of the retrospective design, certain laboratory results were unavailable on admission, including lactate dehydrogenase, d dimer, and serum ferritin and could not be used in the multivariable analysis. Patients with chronic lung disease and conditions associated with immunosuppression were only a small percentage among hospitalized patients. Therefore, the role of some of these variables in predicting the mortality from COVID-19 might have been underestimated. Another limitation of this analysis was not considering 30- or 90-day postdischarge mortality; patients may die soon after discharge related to COVID-19 hospitalization, and that information was not collected as part of the outcome.

In conclusion, our study showed independent predictors for mortality among hospitalized COVID-19 patients by sex, including presence of public insurance type, qSOFA, and elevated CRP in both sexes. Male-specific factors for in-hospital mortality were older age, lymphocytopenia and thrombocytopenia. In female patients, only a higher Charlson score was associated with mortality. Subsequent research involving multiple study sites and with a larger database can further validate the findings of our study.
